# Methyl 3-(3-pyridylmethyl­ene)carbazate

**DOI:** 10.1107/S1600536809045024

**Published:** 2009-10-31

**Authors:** Yu-Feng Li, Hai-Xing Liu, Fang-Fang Jian

**Affiliations:** a Microscale Science Institute, Department of Chemistry and Chemical Engineering, Weifang University, Weifang 261061, People’s Republic of China; b Microscale Science Institute, Weifang University, Weifang 261061, People’s Republic of China

## Abstract

In the crystal of the title compound, C_8_H_9_N_3_O_2_, mol­ecules are linked by N—H⋯N hydrogen bonds, forming *S*(7) chains propagating in [010].

## Related literature

For background to Schiff bases, see: Cimerman *et al.* (1997 ▶).
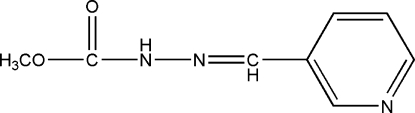


## Experimental

### 

#### Crystal data

C_8_H_9_N_3_O_2_
*M* *_r_* = 179.18
Orthorhombic, 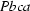

*a* = 10.585 (2) Å
*b* = 10.019 (2) Å
*c* = 16.311 (3) Å
*V* = 1729.8 (6) Å^3^
*Z* = 8
Mo *K*α radiation
μ = 0.10 mm^−1^
*T* = 293 K
0.26 × 0.21 × 0.19 mm

#### Data collection

Bruker SMART CCD diffractometer
Absorption correction: none
15411 measured reflections
1984 independent reflections
1794 reflections with *I* > 2σ(*I*)
*R*_int_ = 0.028

#### Refinement

*R*[*F* ^2^ > 2σ(*F* ^2^)] = 0.046
*wR*(*F* ^2^) = 0.128
*S* = 1.08
1984 reflections
118 parameters
H-atom parameters constrained
Δρ_max_ = 0.24 e Å^−3^
Δρ_min_ = −0.32 e Å^−3^

### 

Data collection: *SMART* (Bruker, 1997 ▶); cell refinement: *SAINT* (Bruker, 1997 ▶); data reduction: *SAINT*; program(s) used to solve structure: *SHELXS97* (Sheldrick, 2008 ▶); program(s) used to refine structure: *SHELXL97* (Sheldrick, 2008 ▶); molecular graphics: *SHELXTL* (Sheldrick, 2008 ▶); software used to prepare material for publication: *SHELXTL*.

## Supplementary Material

Crystal structure: contains datablocks global, I. DOI: 10.1107/S1600536809045024/hb5196sup1.cif

Structure factors: contains datablocks I. DOI: 10.1107/S1600536809045024/hb5196Isup2.hkl

Additional supplementary materials:  crystallographic information; 3D view; checkCIF report

## Figures and Tables

**Table 1:** Hydrogen-bond geometry (Å, °)

*D*—H⋯*A*	*D*—H	H⋯*A*	*D*⋯*A*	*D*—H⋯*A*
N1—H1*A*⋯N4^i^	0.86	2.12	2.9751 (14)	171

Symmetry code: (i) 
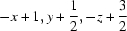
.

**Fig. 1.**
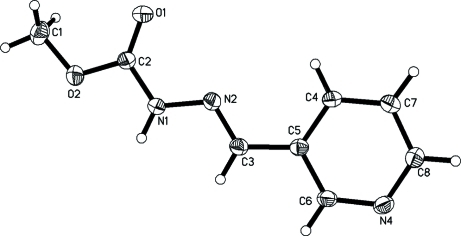
The structure of the title compound showing 30% probability displacement ellipsoids.

## supplementary crystallographic information

### Experimental

A mixture of nicotinaldehyde (0.1 mol), and methyl carbazate (0.1 mol) was stirred in refluxing ethanol (20 mL) for 4 h to afford the title compound (0.082 mol, yield 82%).  Colourless blocks of (I) were obtained by recrystallization from ethanol at room temperature.

### Refinement

H atoms were fixed geometrically and allowed to ride on their attached atoms.

### Crystal data

**Table:**

C_8_H_9_N_3_O_2_	*D*_x_ = 1.376 Mg m^−^^3^
*M**_r_* = 179.18	Mo *K*α radiation, λ = 0.71073 Å
Orthorhombic, *P**b**c**a*	Cell parameters from 1985 reflections
*a* = 10.585 (2) Å	θ = 3.4–27.5°
*b* = 10.019 (2) Å	µ = 0.10 mm^−^^1^
*c* = 16.311 (3) Å	*T* = 293 K
*V* = 1729.8 (6) Å^3^	Block, colourless
*Z* = 8	0.26 × 0.21 × 0.19 mm
*F*(000) = 752	

### Data collection

**Table:**

Bruker SMART CCD diffractometer	1794 reflections with *I* > 2σ(*I*)
Radiation source: fine-focus sealed tube	*R*_int_ = 0.028
graphite	θ_max_ = 27.5°, θ_min_ = 3.1°
ω scans	*h* = −13→13
15411 measured reflections	*k* = −13→12
1984 independent reflections	*l* = −21→21

### Refinement

**Table:**

Refinement on *F*^2^	Primary atom site location: structure-invariant direct methods
Least-squares matrix: full	Secondary atom site location: difference Fourier map
*R*[*F*^2^ > 2σ(*F*^2^)] = 0.046	Hydrogen site location: inferred from neighbouring sites
*wR*(*F*^2^) = 0.128	H-atom parameters constrained
*S* = 1.08	*w* = 1/[σ^2^(*F*_o_^2^) + (0.0867*P*)^2^ + 0.1822*P*] where *P* = (*F*_o_^2^ + 2*F*_c_^2^)/3
1984 reflections	(Δ/σ)_max_ < 0.001
118 parameters	Δρ_max_ = 0.24 e Å^−^^3^
0 restraints	Δρ_min_ = −0.32 e Å^−^^3^

### Special details

**Table:**

Geometry. All e.s.d.'s (except the e.s.d. in the dihedral angle between two l.s. planes) are estimated using the full covariance matrix. The cell e.s.d.'s are taken into account individually in the estimation of e.s.d.'s in distances, angles and torsion angles; correlations between e.s.d.'s in cell parameters are only used when they are defined by crystal symmetry. An approximate (isotropic) treatment of cell e.s.d.'s is used for estimating e.s.d.'s involving l.s. planes.
Refinement. Refinement of *F*^2^ against ALL reflections. The weighted *R*-factor *wR* and goodness of fit *S* are based on *F*^2^, conventional *R*-factors *R* are based on *F*, with *F* set to zero for negative *F*^2^. The threshold expression of *F*^2^ > σ(*F*^2^) is used only for calculating *R*-factors(gt) *etc*. and is not relevant to the choice of reflections for refinement. *R*-factors based on *F*^2^ are statistically about twice as large as those based on *F*, and *R*- factors based on ALL data will be even larger.

### Fractional atomic coordinates and isotropic or equivalent isotropic displacement parameters (Å^2^)

**Table:**

	*x*	*y*	*z*	*U*_iso_*/*U*_eq_	
O2	0.18815 (8)	0.11348 (8)	0.57072 (5)	0.0433 (2)	
C3	0.35300 (9)	−0.17778 (10)	0.74434 (7)	0.0343 (3)	
H3A	0.4286	−0.1375	0.7291	0.041*	
N4	0.48145 (8)	−0.43908 (10)	0.88319 (6)	0.0390 (3)	
C5	0.35393 (9)	−0.28611 (10)	0.80449 (6)	0.0311 (2)	
N1	0.26042 (8)	−0.03820 (9)	0.65555 (6)	0.0372 (2)	
H1A	0.3336	−0.0124	0.6391	0.045*	
C4	0.24493 (9)	−0.34567 (12)	0.83549 (7)	0.0377 (3)	
H4B	0.1656	−0.3156	0.8194	0.045*	
N2	0.24948 (8)	−0.13809 (9)	0.71265 (5)	0.0345 (2)	
C2	0.15392 (9)	0.01936 (10)	0.62537 (6)	0.0339 (3)	
O1	0.04701 (7)	−0.00686 (10)	0.64392 (6)	0.0541 (3)	
C6	0.46946 (9)	−0.33613 (11)	0.83129 (6)	0.0358 (3)	
H6A	0.5428	−0.2957	0.8120	0.043*	
C8	0.37535 (11)	−0.49405 (11)	0.91219 (7)	0.0393 (3)	
H8A	0.3819	−0.5651	0.9486	0.047*	
C7	0.25615 (10)	−0.44962 (12)	0.89025 (7)	0.0414 (3)	
H7A	0.1845	−0.4896	0.9123	0.050*	
C1	0.08717 (14)	0.19038 (13)	0.53648 (8)	0.0523 (3)	
H1B	0.1209	0.2546	0.4987	0.078*	
H1C	0.0430	0.2359	0.5796	0.078*	
H1D	0.0298	0.1322	0.5082	0.078*	

### Atomic displacement parameters (Å^2^)

**Table:**

	*U*^11^	*U*^22^	*U*^33^	*U*^12^	*U*^13^	*U*^23^
O2	0.0420 (5)	0.0450 (5)	0.0429 (5)	0.0031 (3)	0.0027 (3)	0.0115 (3)
C3	0.0289 (5)	0.0385 (5)	0.0355 (5)	−0.0040 (4)	−0.0007 (4)	0.0006 (4)
N4	0.0315 (5)	0.0440 (5)	0.0415 (5)	0.0086 (4)	−0.0001 (4)	0.0016 (4)
C5	0.0289 (5)	0.0341 (5)	0.0302 (5)	−0.0005 (4)	−0.0009 (3)	−0.0034 (4)
N1	0.0277 (4)	0.0426 (5)	0.0413 (5)	−0.0042 (3)	−0.0005 (3)	0.0102 (4)
C4	0.0257 (5)	0.0467 (6)	0.0407 (6)	−0.0008 (4)	−0.0037 (4)	0.0042 (4)
N2	0.0325 (4)	0.0364 (5)	0.0346 (4)	−0.0039 (3)	−0.0018 (3)	0.0033 (3)
C2	0.0324 (5)	0.0363 (5)	0.0330 (5)	−0.0018 (4)	−0.0015 (4)	0.0005 (4)
O1	0.0284 (4)	0.0685 (6)	0.0656 (6)	−0.0020 (4)	−0.0018 (4)	0.0220 (5)
C6	0.0263 (5)	0.0430 (6)	0.0380 (5)	0.0009 (4)	0.0026 (4)	−0.0003 (4)
C8	0.0400 (6)	0.0373 (5)	0.0407 (5)	0.0030 (4)	−0.0004 (4)	0.0038 (4)
C7	0.0312 (5)	0.0465 (6)	0.0463 (6)	−0.0069 (4)	0.0005 (4)	0.0064 (5)
C1	0.0603 (8)	0.0478 (6)	0.0489 (7)	0.0117 (6)	−0.0049 (6)	0.0102 (5)

### Geometric parameters (Å, °)

**Table:**

O2—C2	1.3472 (13)	N1—H1A	0.8600
O2—C1	1.4312 (15)	C4—C7	1.3771 (16)
C3—N2	1.2752 (13)	C4—H4B	0.9300
C3—C5	1.4631 (14)	C2—O1	1.2005 (13)
C3—H3A	0.9300	C6—H6A	0.9300
N4—C8	1.3373 (14)	C8—C7	1.3850 (15)
N4—C6	1.3403 (14)	C8—H8A	0.9300
C5—C6	1.3920 (13)	C7—H7A	0.9300
C5—C4	1.3940 (13)	C1—H1B	0.9600
N1—C2	1.3586 (13)	C1—H1C	0.9600
N1—N2	1.3719 (12)	C1—H1D	0.9600
			
C2—O2—C1	115.73 (9)	O2—C2—N1	108.28 (9)
N2—C3—C5	120.59 (9)	N4—C6—C5	123.96 (9)
N2—C3—H3A	119.7	N4—C6—H6A	118.0
C5—C3—H3A	119.7	C5—C6—H6A	118.0
C8—N4—C6	117.44 (9)	N4—C8—C7	122.77 (10)
C6—C5—C4	117.33 (9)	N4—C8—H8A	118.6
C6—C5—C3	118.92 (8)	C7—C8—H8A	118.6
C4—C5—C3	123.73 (8)	C4—C7—C8	119.29 (10)
C2—N1—N2	119.05 (8)	C4—C7—H7A	120.4
C2—N1—H1A	120.5	C8—C7—H7A	120.4
N2—N1—H1A	120.5	O2—C1—H1B	109.5
C7—C4—C5	119.18 (9)	O2—C1—H1C	109.5
C7—C4—H4B	120.4	H1B—C1—H1C	109.5
C5—C4—H4B	120.4	O2—C1—H1D	109.5
C3—N2—N1	115.47 (8)	H1B—C1—H1D	109.5
O1—C2—O2	124.99 (10)	H1C—C1—H1D	109.5
O1—C2—N1	126.73 (10)		

### Hydrogen-bond geometry (Å, °)

**Table:**

*D*—H···*A*	*D*—H	H···*A*	*D*···*A*	*D*—H···*A*
N1—H1A···N4^i^	0.86	2.12	2.9751 (14)	171

Symmetry codes: (i) −*x*+1, *y*+1/2, −*z*+3/2.
